# Brain Hydrophobic Peptides Antagonists of Neurotoxic Amyloid β Peptide Monomers/Oligomers–Protein Interactions

**DOI:** 10.3390/ijms241813846

**Published:** 2023-09-08

**Authors:** Carlos Gutierrez-Merino

**Affiliations:** Instituto de Biomarcadores de Patologías Moleculares, Universidad de Extremadura, 06006 Badajoz, Spain; biocgm@gmail.com or carlosgm@unex.es

**Keywords:** amyloid β, Alzheimer’s disease, neurotoxicity, endogenous hydrophobic peptides, proteasome, canonical antigen peptides, neuropeptides, calmodulin

## Abstract

Amyloid β (Aβ) oligomers have been linked to Alzheimer’s disease (AD) pathogenesis and are the main neurotoxic forms of Aβ. This review focuses on the following: (i) the Aβ(1–42):calmodulin interface as a model for the design of antagonist Aβ peptides and its limitations; (ii) proteolytic degradation as the major source of highly hydrophobic peptides in brain cells; and (iii) brain peptides that have been experimentally demonstrated to bind to Aβ monomers or oligomers, Aβ fibrils, or Aβ plaques. It is highlighted that the hydrophobic amino acid residues of the COOH-terminal segment of Aβ(1–42) play a key role in its interaction with intracellular protein partners linked to its neurotoxicity. The major source of highly hydrophobic endogenous peptides of 8–10 amino acids in neurons is the proteasome activity. Many canonical antigen peptides bound to the major histocompatibility complex class 1 are of this type. These highly hydrophobic peptides bind to Aβ and are likely to be efficient antagonists of the binding of Aβ monomers/oligomers concentrations in the nanomolar range with intracellular proteins. Also, their complexation with Aβ will protect them against endopeptidases, suggesting a putative chaperon-like physiological function for Aβ that has been overlooked until now. Remarkably, the hydrophobic amino acid residues of Aβ responsible for the binding of several neuropeptides partially overlap with those playing a key role in its interaction with intracellular protein partners that mediates its neurotoxicity. Therefore, these latter neuropeptides are also potential candidates to antagonize Aβ peptides binding to target proteins. In conclusion, the analysis performed in this review points out that hydrophobic endogenous brain neuropeptides could be valuable biomarkers to evaluate the risk of the onset of sporadic AD, as well as for the prognosis of AD.

## 1. Introduction

**Figure 1:**
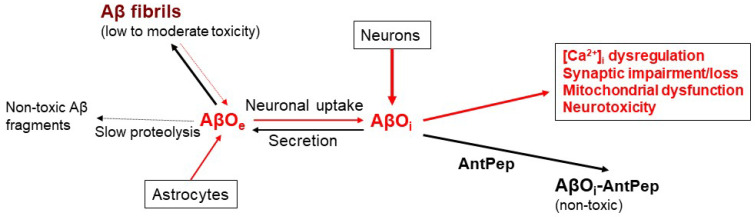
Scheme of Aβ oligomers (AβO) dynamics and neurotoxicity. AβO_e_ and AβO_i_ means AβO extracellular and intracellular, respectively. Generation of neurotoxic AβO are marked by red arrows. Black arrows are used for molecular mechanisms that attenuate the toxicity of AβO, stressing the protection that can be afforded by their complexation with endogenous antagonist Aβ peptides (AntPep).

Amyloid plaques are a neuropathological feature in Alzheimer’s disease (AD) [1]. The prevalent amyloid β (Aβ) peptide found in the amyloid plaques of human AD-affected brains is Aβ(1–42) [2]. Although Aβ plaques are cytotoxic, it has been proposed that Aβ plaques could serve as reservoirs for the assembly of small Aβ oligomers [3], which have been linked to AD pathogenesis and are the main neurotoxic forms of Aβ [4,5,6,7,8,9]. Indeed, Aβ oligomers have been reported to be the primary pathogenic forms of Aβ, which change the structure of synapses and eventually disrupt neuronal communication [10] (see the schematic diagram of Figure 1). Moreover, intraneuronal Aβ accumulation precedes the appearance of amyloid plaques or tangles in transgenic mice models of AD [6,11,12,13]. Aβ(25–35) has been suggested to be the more biologically active region of Aβ(1–42) [14,15] because it is the shortest peptide that retains the toxicity of the full-length Aβ(1–42) peptide [16]. But Aβ is a peptide whose physiological functions are still under study. In a seminal study with neuronal cultures, Yankner et al. [17] found that Aβ(1–40) was neurotrophic to undifferentiated hippocampal neurons at low concentrations and neurotoxic to mature neurons at higher concentrations.

The mechanism of amyloid plaque formation suggests an intracellular basis of Aβ pathogenicity [9]. The plaques of Aβ fibrils in AD are extracellular and increasingly being viewed as innocuous sinks for misfolded Aβ because amyloid fibril formation is also related to essential biological functions, like protein replication, the storage of peptide hormones and mammalian skin pigmentation [18,19]. Furthermore, it has been suggested that it is an antimicrobial peptide [20]. Amyloid fibrils that perform physiological roles are called functional amyloids and are not generated only by Aβ peptides. For example, tachykinin neuropeptides, which have a COOH-terminus (Phe–X–Gly–Leu–Met–NH_2_, where X is an aromatic or aliphatic residue) similar to Aβ(25–35), are one of the functional amyloids and perform diverse functions, such as exciting neurons, evoking behavior responses, and contracting smooth muscle [21,22]. On the other hand, cross-interactions between different amyloidogenic proteins or polypeptides, “cross-amyloid interactions”, modulate their self-assembly into amyloid fibrils and may link different diseases to each other [23,24,25]. Luo et al. [26] proposed that the amyloid cascade hypothesis in Alzheimer’s disease should be expanded to include cross-interactions between Aβ and other amyloid proteins, like tau, human prion protein (PrP^C^), α-synuclein, and other proteins present in the cerebrospinal fluid during various disease conditions, such as lysozyme, transthyretin, apolipoprotein A1, and blood proteins like serum amyloid P component and fibrinogen.

In human AD-affected brains, Aβ monomers/oligomers can be produced in neurons or in astrocytes, which also secrete neurotoxic Aβ peptides [27]. Indeed, it has been reported that the percentage of NH_2_-terminally truncated Aβ secreted by astrocytes is much higher than that of Aβ secreted by neurons, i.e., 60% and 20%, respectively [28], and a large fraction of the Aβ species present in Aβ plaques are NH_2_-terminus truncated [29,30]. Plasma membrane lipid rafts have been shown to play an active role in extracellular Aβ uptake and internalization in neurons, reviewed in [31]. Aβ peptides interact with cholesterol and gangliosides in ganglioside-clustered raft-like membrane microdomains, which potentiate the formation of Aβ oligomers and fibrils in a cholesterol-dependent manner [32,33,34,35,36]. Particularly relevant among intracellular targets of Aβ(1–42) oligomers are neuronal non-amyloidogenic proteins showing high affinity for nanomolar concentrations of Aβ peptides, because critical concentration values in the sub micromolar range have been reported for the induction of Aβ(1–42) fibrillization [37,38], and concentrations of non-fibrillar Aβ peptides within the nanomolar range have been reported in the brain [39,40,41]. Among these proteins, the dissociation constant of Aβ(1–42) has been reported to be around 1 nM only for tau [42], cellular prion protein (PrP^C^) [43], glycogen synthase kinase 3α (GSK3α) [44], calmodulin (CaM) [45], and likely stromal interaction molecule 1 (STIM1) [46]. Human PrP^C^ is a glycoprotein that largely localizes to cholesterol-rich lipid rafts on the outer surface of the cell membrane, thereby acting as a high-affinity receptor for extracellular Aβ oligomers in concert with the low-density lipoprotein receptor-related protein-1 [47]. However, in neurons the concentration of CaM is In the micromolar range [48,49], which is orders of magnitude higher than the concentration of the other competing proteins in the neurons listed above. Due to this, CaM can be considered a major sink for neurotoxic intracellular Aβ peptides, and this in turn suggests that CaM could play a key role in protecting against an increase in free intracellular Aβ concentrations above 1–2 nM [50]. Furthermore, Aβ(1–42):CaM complexes can also function as intracellular transducers for focalized actions of Aβ peptides due to the many roles of CaM in Ca^2+^-signaling pathways modulating neuronal metabolism, excitability, and cell death known to be altered in AD [50]. The complexation between CaM and Aβ peptides is driven by interactions involving exposed hydrophobic domains of CaM and hydrophobic amino acids of Aβ [45,51] and produces structural changes in CaM [52].

The next sections of this review deal with the following: (i) the Aβ(1–42):CaM interface as a model for the design of antagonist Aβ peptides and its limitations; (ii) proteolytic degradation as the major source of highly hydrophobic peptides in brain cells; and (iii) brain peptides that have been experimentally demonstrated to bind to Aβ monomers or oligomers, Aβ fibrils, or Aβ plaques.

## 2. The Aβ(1–42):CaM Interface as a Model for the Design of Antagonist Aβ Peptides and Its Limitations

In a previous work [51], the amino acid residues of Aβ(1–42) and CaM at the interface of the complex formed between both molecules were obtained using protein docking approaches with their three-dimensional structures, which are available in the UniProt protein data bank. The analysis led to the conclusion that this interface is strongly hydrophobic. The interacting interface of the two most probable simulations of the Aβ(1–42):CaM complex compatible with our experimental results reported in [45] was analyzed using PDBePISA (Protein Interfaces, Surfaces, and Assemblies). This analysis yielded very high values of buried surface area/accessible surface area ratio (BSA/ASA), scoring between 0.8 and the maximum value of 1.0, for the following hydrophobic amino acid residues of Aβ(1–42): Gly37 (1.0); Gly38 (0.98–1.0); Ala42 (0.99); Val36 (0.98); Phe20 (0.97); Met35 (0.96); Val40 (0.89); Leu34 (0.83); Val39 (0.82–0.99); and Ile41 (0.82). Therefore, the strongly hydrophobic segment comprising the amino acids residues 34–42 of the Aβ(1–42) plays a major role in the formation of the Aβ(1–42):CaM complex. Also, it was noticed in [51] that the interface of the complex formed between Aβ(1–42) and calbindin-D28k is strongly hydrophobic, with high BSA/ASA for most of the above-listed amino acids, plus high score ratios for other hydrophobic amino acid residues of Aβ(1–42), namely Val24 (1), Phe20 (0.86–0.98), Val18 (0.88), Ile31 (0.84–0.91), Leu17 (0.75–0.91), Gly33 (0.62–0.98), Ala21 (0.65), Ile41 (0.63), and Ala30 (0.55–0.75). An interface domain for the Aβ(1–42):calbindin-D28k complex larger than that for the Aβ(1–42):CaM complex is an expected result because the size of calbindin-D28k is larger than that of CaM. However, it is to be noted that in both cases, the interfaces between the complexes of Aβ(1–42) with these proteins are strongly hydrophobic and comprise most of hydrophobic amino acid residues of the COOH-terminus domain of Aβ(1–42). Interestingly, fragment sequences derived from the COOH-terminal section of Aβ(1–42) have been found to directly interact with full-length Aβ peptides and inhibit fibril formation and toxicity [53]. Furthermore, Aβ(25–35) has been reported to show the same early neurotrophic and late neurotoxic activities as Aβ(1–40), while Aβ(1–16) and Aβ(17–28) showed no trophic or toxic activity at 20 μM [17].

In order to design a hydrophobic peptide that could antagonize the formation of the Aβ(1–42):CaM complex, in [51], analysis of the hydrophobic amino acid residues of CaM which are in close proximity with the above-listed Aβ(1–42) amino acid residues in the predicted interface of the Aβ(1–42):CaM complex was performed, as well as analysis of those that scored with values of BSA/ASA higher than 0.8 in the most probable structures of this complex generated in silico by the ClusPro server. The peptide VFAFAMAFML (amidated-C-terminus amino acid), which mimics the interacting domain of CaM with Aβ (1–42) predicted by docking, was experimentally shown to antagonize the complexation between CaM and calbindin-D28k with a fluorescent derivative of Aβ(1–42) [51]. Only sub micromolar to micromolar concentrations of this peptide were found to afford a nearly complete blockade of the formation of complexes between Aβ(1–42) and CaM or calbindin-D28k.

**Figure 2:**
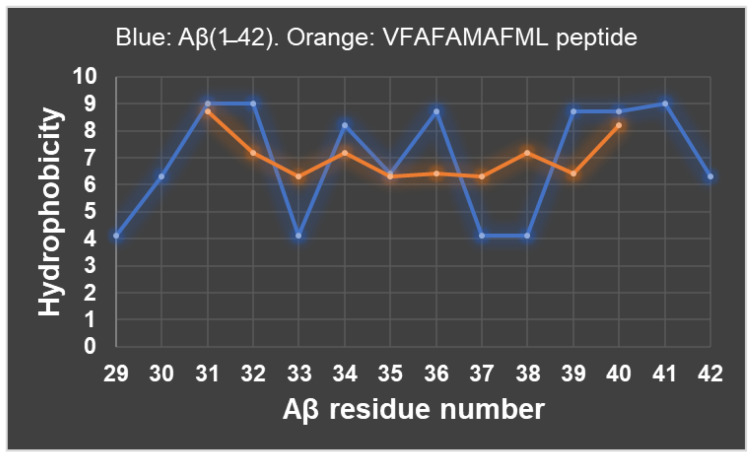
Hydrophobicity plot of Aβ(1–42) segment 29–42 (blue) and of the designed peptide VFAFAMAFML (orange). The NH_2_-terminus amino acid residue of the peptide VFAFAMAFML has been aligned with the amino acid residue 31 of Aβ(1–42).

The peptide VFAFAMAFML (amidated-C-terminus amino acid) is the first peptide antagonist of Aβ(1–42) to be generated by rational design. Also, it is to be recalled that CaM and calbindin-D28k are the proteins expressed at the highest levels in brain neurons among those that bind neurotoxic Aβ peptides with dissociation constants in the nanomolar range [51], i.e., these are the major sinks for trapping intracellular nanomolar concentrations of neurotoxic Aβ peptides in these cells. In addition, it is likely that the amino acid residues of Aβ(1–42) interacting with these proteins play a critical role in its complexation with other proteins that have a similar high affinity for Aβ(1–42). Also, it is to be noted that, using the standard hydrophobicity values reported for amino acids [54], many possible peptides of alternate hydrophobic amino acids sequences can be generated with close hydrophobicity to that of VFAFAMAFML (amidated-C-terminus amino acid) (Figure 2). Moreover, using the hydrophobic compatibility matrix proposed in [55], a large number of alternate interacting hydrophobic amino acids forming pairs with residues 34 to 42 of the Aβ(1–42) yields a compatibility index higher than 80%, for example, Leu-Leu, Leu-Ile, Val-Val, Phe-Phe, Phe-Val, Phe-Leu, Phe-Ile, Phe-Met, Phe-Ala, Met-Ala, Met-Leu, Met-Ile, Ala-Ala, Ala-Leu, Ala-Val, and Ala-Ile. This number can be reduced taking into account the need of size compatibility of the lateral side chains of amino acids in the interface of the Aβ(1–42):protein complex, as is usually the case in the three-dimensional predictions of protein structure [55], but many alternate compatible amino acid sequences still remain. For example, proline-rich hydrophobic peptides have been found to alter Aβ(1–42) folding and fibril formation [56]. Also, the reported structural plasticity of the COOH-terminus domain of Aβ(1–42) [57] is another factor that does not contribute to achieve a large reduction in alternate peptides with hydrophobic compatible amino acid sequences. Therefore, it can be anticipated that a large number of small peptides with alternate sequences of hydrophobic amino acids are expected to behave as antagonists of Aβ(1–42).

A priori, it can be predicted that Aβ(1–42) should bind with high affinity to proteins with a three-dimensional exposed patch of 8–10 lateral side chains of strongly hydrophobic amino acid residues with a high size compatibility with the amino acid residues 34–42 of Aβ(1–42). Indeed, this seems to be the case for the proteins with high affinity for nanomolar concentrations of Aβ(1–42) listed above in this article, as briefly summarized next. Hydrophobic surfaces between β-sheet layers are important in inhibiting amyloid aggregation, and a macrocyclic β-sheet peptide inhibits the aggregation of the tau-protein-derived peptide Ac-VQIVYK-NH_2_ [58]. Moreover, it has been shown that liquid–liquid phase separation of tau driven by hydrophobic interaction facilitates fibrillization of tau [59]. Also, a hydrophobic site that binds axin and adenomatous polyposis coli protein has been localized in the C-terminal helical domain of GSK3 [60,61]. The scaffold protein axin binds the transcriptional co-activator β-catenin [62], and the level of β-catenin hyperphosphorylation by GSK3 plays a key role in Wnt signaling [61,63]. Dajani et al. [61] identified the axin-derived 19 residue peptide that binds as a single amphipathic α-helix into a hydrophobic surface channel on the COOH-terminal domain of GSK3. In STIM1, the EF-hands of two monomers form a hydrophobic cleft that binds to hydrophobic residues in the sterile-α-motif domain in order to stabilize the resting state of the structure [64]. The drop of Ca^2+^ in the endoplasmic reticulum elicits the unfolding of the EF-sterile-α-helix domain, leading to exposure of hydrophobic surfaces that trigger the aggregation of STIM proteins into dimers and higher-order oligomers in solution [65,66], which interact and activates Orai channels [64]. STIM1 are assumed to be dimers before store depletion, and the interaction of STIM1-COOH-terminal fragment monomers is mediated via several hydrophobic and hydrogen bond interactions [67]. Wang et al. [68] showed that Aβ(1–42) binds to the α7 nicotinic acetylcholine receptor with high affinity. The formation of the complex between α7 nicotinic acetylcholine receptor and Aβ(1–42) can be efficiently suppressed by Aβ(12–28), implying that this Aβ sequence region contains the binding epitope [68]. Since most of the hydrophobic surface of membrane proteins is located at the lipid–protein interface, the lipid microenvironment is likely to play a structural role in high-affinity binding sites of Aβ(1–42) in membrane proteins.

The most prevalent genetic risk factor in nonfamilial AD is the ε4 allele of the gene-encoding apolipoprotein E (apoE), and it has been proposed that some apolipoproteins act as soluble chaperones for hydrophobic peptides, such as Aβ [69]. Indeed, it has been shown that apoE2 and apoE3 bind soluble Aβ, and apoE4 preferentially binds to an intermediate aggregate form of Aβ [70,71]. The immunoreactivity of apoE correlates with that of intracellular Aβ in AD brain samples, and it has been suggested that apoE is internalized with Aβ [72,73]. Of note, the low-density lipoprotein receptor-associated protein, an antagonist of this receptor, also forms complexes with soluble Aβ like apoE and promotes its cellular uptake [74]. ApoE accumulates in lipid rafts in transgenic mice, suggesting that the apoE-Aβ complex may target raft-associated receptor proteins [75]. For example, apoE has been observed to target neurotransmitter receptors like the α7 nicotinic acetylcholine receptor [76]. This has led to the hypothesis that uptake of Aβ by neurotransmitter receptors may be due to apoE-receptor binding rather than due to direct interaction between Aβ and the receptor [31]. The aggregation promoting effect of the complexation of GM1 with Aβ has been proposed to account, at least in part, for binding of Aβ to lipid rafts and seeding for subsequently fibril formation [34,77,78,79]. In addition, cholesterol, which is another lipid enriched in lipid rafts, has also been shown to interact with soluble and fibrillar Aβ [35].

The high-affinity binding of Aβ(1–42) to human PrP^C^ merits a special comment because this protein has also a very high affinity for the transition metal ions Cu^2+^ and Zn^2+^ [80], like Aβ(1–42). In PrP^C^, the critical regions for the interaction with Aβ(1–42) are the ~95–110 segment and a cluster of basic residues at the extreme NH_2_-terminus of PrP^C^ (residues 23–27) [43,81]. Since the ~95–110 segment of PrP^C^ partially overlaps with the octa repeat-containing flexible tail that binds Cu^2+^ [82], it is likely that transition metal ions play a major role in the interaction between Aβ(1–42) and human PrP^C^.

Therefore, the design of Aβ antagonist peptides that are specific for them should also consider particular structural motifs of the selected protein.

## 3. Proteolytic Degradation as the Major Source of Highly Hydrophobic Peptides in Brain Cells

Highly hydrophobic sequences of amino acid residues are largely compartmented in the interior of globular domains of proteins during the protein-folding process. Proteolysis is likely the main intracellular source of 8–10 amino acid sequences with a hydrophobicity similar to that of the Aβ(1–42) antagonist peptide VFAFAMAFML (amidated-C-terminus amino acid). Thus, the possibility that the trapping of highly hydrophobic peptides of 7–10 amino acid residues released during proteolysis could be a physiological function of Aβ emerges, which has been overlooked until now. Indeed, this peptide size is close to the average size of peptides released after protein digestion in the mammalian proteasome [83]. Indeed, the proteasome generates the bulk of antigenic peptides of 8–10 residues long presented by major histocompatibility complex (MHC) class I molecules [83,84,85]. These peptides escape complete degradation and are transported into the endoplasmic reticulum, where they bind the MHC class I molecules [83,86,87]. The endoplasmic reticulum peptide transporter has broad peptide specificity, and MHC class I molecules select a limited set of peptides among those transported into the endoplasmic reticulum lumen, which are the canonical peptides for presentation at the cellular surface [88]. Yet, the canonical antigen peptides show a high content and short sequences of hydrophobic aliphatic and aromatic amino acids, which are key residues for binding to antigen pockets in MHC class I molecules (see, for example, [88,89,90]). Also, it is to be noted that mammalian proteasomes can release peptides of up to 22 residues, which are further degraded by cytosolic endopeptidases [91]. Since Aβ(1–42) is not a good substrate for these cytosolic endopeptidases, the complexation of these peptides with Aβ(1–42) can be seen as a protection mechanism against their rapid degradation in the cytosol, unveiling a “chaperon-like” role of Aβ(1–42) that has not been previously noticed.

However, the complexation by Aβ(1–42) of hydrophobic peptides released from the proteasome have a dual role since these peptides could act as endogenous antagonists of the neurotoxicity of Aβ peptides. Thus, a decline in the activity of the proteasome will lead to an increase in intracellular Aβ(1–42) available for other intracellular targets. Indeed, the proteasome activity has been shown to decrease in brains from AD patients compared with age-matched controls [92], and the inclusion bodies of AD contain abnormal amounts of ubiquitin, providing an additional evidence of proteasome dysfunction in AD neurodegeneration [93]. In the case of AD, it has also been reported that intracellular Aβ oligomers inhibit proteasome activity [94]. These results are in line with the decline in proteasome function during aging and senescence observed in the brain regions more prone to neurodegeneration and other tissues [95,96,97,98]; they also lend support to the hypothesis that this decrease can trigger the onset of age-related diseases [99]. AD is characterized by the deposition of extracellular senile (amyloid) plaques and intracellular neurofibrillary tangles [100]. Clearly, AD is an aging-associated gain-of-toxic-function disease, in which aggregation-mediated proteotoxicity exceeds the cellular clearance machinery [100,101,102,103,104].

The steady state level of Aβ, a physiological peptide, is maintained by the balance between the anabolic and catabolic activities [40,105,106]. The protease neprilysin, a neutral endopeptidase, has been shown to play a rate-limiting role in Aβ catabolism [107,108,109,110]. Neprilysin is a membrane-bound zinc metalloprotease ectoenzyme, with the active site facing the extracellular side of the plasma membrane [111,112]. It has been demonstrated to be the major Aβ-degrading enzyme in the brain, and its expression is reduced by 50–70% in the hippocampus and mid-temporal gyrus, i.e., in the brain regions displaying high amyloid plaques load of sporadic AD patients with respect to age-matched controls [107,110,113]. Thus, neprilysin plays a relevant role in the clearance of extracellular Aβ peptides in the brain regions more severely affected in AD. A closely related protease, neprilysin-like endopeptidase (NEP2), also degrades Aβ peptides efficiently in the brain, albeit with a regional distribution more restricted than that of neprilysin, and its activity is reduced in AD patients compared to non-impaired individuals [114]. Other proteases that have been shown to significantly contribute to the degradation of extracellular Aβ peptides are the endothelin-converting enzymes 1 and 2 (ECE-1 and ECE-2) [115,116]. But the proteases that degrade cytosolic Aβ peptides are still a matter of debate. ECE-2 has been shown to be associated with intracellular membranes, but the optimum pH of its activity is 5.0–5.5 [116], pointing out that it is likely involved in the clearance of Aβ peptides within acidic subcellular organelles like lysosomes but not in the cytosol. Puromycin-sensitive aminopeptidase overexpression reduces Aβ levels and toxicity in *Drosophila*, but this is an effect which has been reported to be independent of its proteolytic activity [117]. It has been noted that the mechanism via which this occurs is unknown as this aminopeptidase does not degrade Aβ in vitro [118]. On the other hand, a low rate of cytosolic degradation of Aβ peptides places a stringent requirement to control its production, which takes place in this subcellular compartment, to prevent them accumulating and reaching a cytotoxic concentration range. Under these cellular conditions, the capping of Aβ(1–42) and shorter neurotoxic Aβ peptides like Aβ(1–40) and Aβ(25–35) with hydrophobic peptides released from the proteasome could serve as a defense mechanism to prevent their interaction with cytosolic molecular targets that mediate their toxic effects.

## 4. Brain Peptides That Have Been Experimentally Demonstrated to Bind to Aβ Monomers or Oligomers, Aβ Fibrils, or Aβ Plaques

A plethora of neuropeptides and other peptides present in the cerebrospinal fluid play major roles in normal brain functioning. Alterations of the level of various neuropeptides and of their receptors have been reported in the brain of AD patients [119,120] and of brain peptides according to global neuropeptidomic analysis [121]. Many neuropeptides have been reported to exert neuroprotective actions against brain degeneration in AD (see, for example, the reviews in [119,120,122,123]). Neuroprotective peptides in AD are widely present in the brain areas responsible for learning and memory processes. Only peptides present in the human brain, for which experimental data have been reported in terms of their interaction with neurotoxic Aβ peptides, are the focus of this review and are dealt with in this section.

In a pioneer work, Yankner et al. [17] found that tachykinin neuropeptides substance P and physalaemin can inhibit both the early neurotrophic and late neurotoxic effects of Aβ(1–40) in hippocampal neurons with an inhibitory 50% concentration lower than 1 μM. Neurokinin B was found to be less potent and to be partially inhibitory at micromolar concentrations, and other tachykinins like neurokinin A, eledoisin, and kassinin were found to have not significant effects up to 20 μM. These authors noticed a high homology between the sequences of tachykinin peptides and that of Aβ(25–35). Indeed, all tachykinin peptides have a COOH-terminus Phe–X–Gly–Leu–Met–NH_2_, where X is an aromatic or aliphatic residue, similar to Aβ(25–35) [21]. Since the Aβ(1–40) effects were mimicked by antagonists of tachykinin receptors, the authors rationalized their experimental data in terms of Aβ(1–40) binding to these receptors [17]. Substance P has been found in Aβ plaques of patients with Alzheimer’s disease [124,125], a result that suggests strong interactions between Aβ and substance P. Later, coincubation studies between kassinin and Aβ(25–35), as well as between substance P and Aβ(25–35), reported to foster Aβ aggregation and fibrils formation [126], but a computational study predicts that neurokinin B should inhibit the formation of Aβ(25–35) dimers [127]. More recently, Liu et al. [22] reported that neurokinin B and substance P remove the Aβ(25–35) hexamers and dodecamers, which are related to its toxicity, although substance P did so more slowly, and, in contrast, kassinin was found to promote the formation of these higher-order oligomers. As noted in [22], these results are somewhat at odds with the literature data that suggest all three peptides are protective against Aβ neurotoxicity. Further experimental work is needed to clarify the relative relevance of Aβ-tachykinin complexation and of Aβ-tachykinin receptor interaction in the protection against Aβ neurotoxicity.

Soper et al. [128] reported that leucine enkephalin and galanin interact both with the monomeric and small oligomeric forms of Aβ(1–40), with the interaction with leucine enkephalin being stronger than that of galanin and yielding a range of complexes with diverse stoichiometries. These authors identified a region of Aβ between its NH_2_-terminal tail and hydrophobic core of Aβ(1–40) directly implicated in the noncovalent binding of leucine enkephalin. They noted that this is a region of Aβ(1–40) similar to that shown to bind (–)-epigallocatechin-3-gallate in a previous work [129], a natural product that has been reported to inhibit Aβ fibril formation and neurotoxicity [130,131]. Nevertheless, the biological significance of the interaction of leucine enkephalin with Aβ should be taken cautiously at present because the reported dissociation constant of the leucine enkephalin:Aβ monomer is ~60 μM, measured in the absence of metal ions, which is orders of magnitude higher than that reported for leucine enkephalin from opioid receptors [132]. In addition, no Aβ complexes were detected for substance P, somatostatin, or neurotensin, even when added in large excess in solution [128]. Since the result obtained with substance P was somewhat controversial with other works (see above), the authors argued that it “either interacts with larger toxic oligomers that are not detected in our ion mobility-mass spectrometry datasets, or that the action of substance P is related to its role as a neuronal agonist, where it may act to block Aβ interactions with critical cell surface receptors”.

More extensive identification of Aβ amino acids residues involved in the interaction with a peptide present in the cerebrospinal fluid has been performed with the intrinsically disordered polypeptide islet amyloid polypeptide (IAPP), which is associated with type 2 diabetes [23,25]. A nanomolar affinity interaction between early prefibrillar Aβ(1–40) and IAPP species has been shown in vitro to suppress amyloidogenesis [133], whereas seed amounts of Aβ(1–40) fibrils are able to cross-seed IAPP amyloidogenesis in vitro and in animal models in vivo [23,133,134,135,136]. Furthermore, IAPP has been reported to co-localize with Aβ plaques in human AD-affected brains, suggesting a possible pathophysiological role for the cross-interaction between the two polypeptides [23,137,138]. Andreeto et al. [139] identified hot regions of the Aβ–IAPP interaction interface as high-affinity binding sites in both cross- and self-association. The hydrophobic COOH-terminal part Aβ(29–40) plays a crucial role in the Aβ(1–40)–IAPP interaction [135]. Andreeto et al. [139] performed an extensive screening of short peptides using membrane-bound peptide arrays of 10-residue Aβ(1–40) and IAPP sequences covering full-length Aβ(1–40) and IAPP and positionally shifted by one residue. Their results yielded Aβ(29–40), Aβ(25–35), and Aβ(35–40) as the 10-residue Aβ peptides that are the stronger ligands for IAPP, with apparent dissociation constants of 200, 282, and 354 nM, respectively, which are only between 4 and 7-fold higher than the 48.5 nM value obtained for Aβ(1–40). Moreover, the analysis of the Aβ(1–40) regions involved in hetero-association with IAPP and in Aβ(1–40) self-association suggested common molecular recognition features in amyloid self- and cross-amyloid hetero-assembly [139]. Later, Yan et al. [133] identified single aromatic/hydrophobic residues within the IAPP amyloid core region that are able to control its interaction with Aβ(1–40). Bakou et al. [25] identified four aromatic/hydrophobic residues of IAPP, which, in combination, are able to control both IAPP amyloid self-assembly and its cross-interaction with Aβ(1–40) and Aβ(1–42).

The Aβ(1–42) interactome using biotinylated monomeric or oligomeric Aβ(1–42) peptides as baits and human frontal lobes as the biological source material uncovered the small cyclic neuropeptide somatostatin (SST) to be the most selectively enriched binder to oligomeric Aβ(1–42) [140,141]. Wang et al. [140] found that somatostatin-14 (SST14) slows down Aβ aggregation and promotes the formation of Aβ assemblies with a 50–60 kDa sodium dodecyl sulfate-resistant core. Moreover, the coincubation of Aβ(1–42) and SST14 led exclusively to oligomeric assemblies [141]. Solarski et al. [141] noted that the ‘NFFWK’ core Aβ-binding epitope within SST bears resemblance to the ‘LVFFA’ segment within Aβ (residues 17–21), which is considered a critical determinant for Aβ fibrillogenesis and has served as a template for derivatizing effective β-sheet breaker peptides [142]. SST14 has been shown to be stored as amyloid in dense core secretory granules prior to its regulated synaptic release [19] and has been shown to acquire amyloid properties in vitro [143]. Notably, an accelerated reduction in SST immunoreactivity has been one of the earliest biochemical changes reported in the cerebral cortex of AD patients [144]. Since SST induces the release of Aβ-degrading enzymes, declining levels of SST, observed during aging and more accentuated in AD [145], may be responsible for reduced clearance of Aβ, leading to its net accumulation and, eventually, Aβ-induced cell death in AD [146]. Therefore, monomeric SST is expected to act in a dual protective manner due to its ability to induce the release of Aβ-degrading enzymes and to interfere with Aβ fibrillization.

Hormone insulin has been shown to display a high probabilistic sequence consistency with the NH_2_-termini and the COOH-termini of amyloid proteins in multiple alignment of amyloid protein sequences calculated by the T-coffee web server [26]. Also, it has been reported that monomeric insulin interacts with soluble Aβ in vitro, inducing the formation of less toxic Aβ oligomers [147]. Although this could account, at least in part, for some biological effects of Aβ, such as Aβ inhibiting the effect of insulin on the secretion of Aβ precursor protein and competing with insulin for binding to the insulin receptor [148], there is a lack of experimental data supporting insulin complexation with Aβ in vivo.

In addition, several neuropeptides have been reported to be associated with Aβ plaques, although it must be noted that the experimental determination of their dissociation constants from Aβ monomers or oligomers and the identification of the amino acid residues of Aβ interacting with these neuropeptides are still pending issues. These neuropeptides are listed in the next paragraphs:

(1) The neuropeptide 7B2 (212 amino acids) has been demonstrated to efficiently prevent in vitro fibrillation and formation of Aβ aggregates, and that recombinant 7B2 protected against the Aβ(1–42)-induced loss of cell viability of Neuro-2A cells [149]. The authors hypothesized that this neural protein could act as an Aβ antiaggregating chaperone in neurodegenerative diseases. In this article, it is also shown that 7B2 highly co-localizes with Aβ plaques in the hippocampus and substantia nigra of human AD-affected brains, as well as in the brains of Aβ precursor protein/presenilin-1 transgenic mice [149];
(2) The recombinant neuropeptide ProSAAS (260 amino acids) and its endogenously produced ProSAAS fragment 97–180 have been shown the prevent the fibrillation of Aβ(1–42) in Neuro2a cells, as well as Aβ(1–42) neurotoxicity to these cells [150]. Moreover, these authors reported that ProSAAS co-localizes with Aβ plaques deposits in the cortex of the AD-affected brain.
(3) Other neuropeptides for which its co-localization with Aβ plaques has been shown using immunostaining of post mortem brain samples of human AD-affected brains are the cocaine- and amphetamine-regulated transcript encoded peptides (40–47 amino acids) [151] and chromogranin A and B-derived peptides [152,153].

Finally, the mitochondria is an alternate source of peptides in AD since mitochondria dysfunction has been linked to metabolic and oxidative damage in this disease [154,155]. Two of these peptides, humanin and small humanin-like peptide 2, have been reported to protect against Aβ toxicity [156,157,158]. Romeo et al. [159] reported that humanin interacts with Aβ oligomers and counteracts Aβ in vivo toxicity, and others have shown that the humanin level in cerebrospinal fluid is lowered in AD patients relative to age-matched controls [160]. Small humanin-like peptide 2 binds IAPP species and blocks amyloid seeding [158].

## 5. Conclusions

This review highlights that many experimental data support the notion that the hydrophobic amino acid residues of the COOH-terminal segment of Aβ(1–42) play a key role in its interaction with intracellular protein partners linked to its neurotoxicity. Also, it is shown that there is a large number of brain peptides with the potential to act as antagonists of the neurotoxic Aβ peptides’ interaction with target intracellular proteins. The analysis of published data conducted in this review allows one to predict that highly hydrophobic peptides of 8–10 amino acids will act as efficient antagonists of the binding of nanomolar concentrations of Aβ monomers/oligomers with intracellular proteins. These amino acid sequences are usually present in the inner core of many proteins; therefore, the proteasome activity is likely the major source of this type of endogenous peptides in neurons. Since many canonical antigen peptides bound to MHC class 1 are also highly hydrophobic peptides of 8–10 amino acids, their complexation with Aβ suggest a putative chaperon-like physiological function for Aβ that has been overlooked until now. Interestingly, proteasome activity is increased in long-lived humans (centenarians) [161]. Thus, improving the proteasome activity in AD patients should be expected to attenuate the neurotoxic actions of Aβ monomers/oligomers.

In addition, a relatively large number of neuropeptides that have been experimentally shown to bind Aβ monomers/oligomers affords neuroprotection against the toxic actions of Aβ on neurons, but the Aβ-interacting amino acid residues are known for only several of them. Table 1 highlights their association with Aβ plaques in AD-affected brains and the reported changes in their expression level in the cerebrospinal fluid or key brain regions in AD relative to patients relative to age-matched controls. In these cases, there is, at least, a partial overlap between the hydrophobic amino acid residues of Aβ responsible for the binding to these neuropeptides and those that play a key role in its interaction with intracellular protein partners that mediate its neurotoxicity. Therefore, these latter neuropeptides are also potential candidates to antagonize Aβ peptides binding to target proteins. Global neuropeptidomic analysis of AD-affected brain samples have started to reveal significant differences with age-matched individuals. Despite the fact that AD is a multifactorial disease, as noted above in this review, Aβ oligomers are recognized to play a major role in the pathogenesis of this disease. Thus, interindividual variation in the level of hydrophobic endogenous neuropeptides that bind Aβ monomers/oligomers is likely to affect the onset of sporadic AD and the rate of brain damage spreading in this neurodegenerative disease. The analyses performed in this review point out that these endogenous brain neuropeptides could be valuable biomarkers to evaluate the risk of the onset of sporadic AD and for the prognosis of AD. Also, this analysis suggests that hydrophobic endogenous brain peptides are candidates to become targets in the development of novel therapies against Aβ-induced neurodegenerative diseases. Regarding this latter point, it is to be emphasized that AD is a multifaceted disease that likely will require multi-therapeutic approaches to be slowed down or eventually kept in a stage of mild cognitive disorder.

## Figures and Tables

**Table 1:** Summary of the main brain endogenous peptides analyzed in this work that can antagonize, at least in part, the neurotoxicity of Aβ peptides through binding Aβ monomers/oligomers.

Endogenous Peptide	Level in the Brain and/or Its Association with Aβ Plaques in AD-Affected Brains
Highly hydrophobic peptides of 8–10 amino acids released by the proteasome, like antigenic MHC class 1 peptides and related peptides	The proteasome activity decreases in brains from AD patients compared with age-matched controls [92]. Decline in proteasome function during aging [95,96,97,98].
Tachykinins like substance P and neurokinin B	Substance P found in Aβ plaques of patients with Alzheimer’s disease [124,125]. Substance P level decreases in cortex, hippocampus, and dentate gyrus of AD patients [162,163]. Elevated levels of substance P in the cerebrospinal fluid of late onset AD patients [164].
IAPP	IAPP co-localizes with Aβ plaques in human AD-affected brains [23,137,138]. Epidemiological and pathophysiological evidences suggest that the AD and type 2 diabetes are linked to each other [23,138].
SST and SST-14	Reduction in SST immunoreactivity in the cerebral cortex of AD patients [144]. SST is the most selectively enriched binder to oligomeric Aβ(1–42) in human frontal lobes [140,141].
Humanin and small humanin-like peptide 2	The level of mitochondrial-derived humanin in cerebrospinal fluid is lowered in AD patients relative to age-matched controls [160].
Neuropeptide 7B2	The neuropeptide 7B2 co-localizes with Aβ plaques in the hippocampus and substantia nigra of human AD-affected brains [149]. Controversial reports on changes in the levels of 7B2 neuropeptide in AD brains [120].
ProSAAS	ProSAAS co-localizes with Aβ plaques in the cortex of AD-affected brain [150]. ProSAAS fragments decrease in the cerebrospinal fluid of AD patients relative to age-matched controls [165].
Cocaine- and amphetamine-regulated transcript encoded peptides	Increased immunoreactivity in the cortex and co-localization with Aβ plaques in post mortem brain samples of human AD-affected brains [151].
Chromogranin A and B-derived peptides	Co-localization with Aβ plaques in post mortem brain samples of human AD-affected brains [152,153]. Decline with time in the cerebrospinal fluid of AD patients [166], and lower levels in the cerebrospinal fluid of mild AD patients relative to cognitive normal controls [167].

## Data Availability

No new data are created.

## Abbreviations

Aβ	amyloid β peptide
AD	Alzheimer’s disease
ApoE	apolipoprotein E
BSA/ASA	buried surface area/accessible surface area ratio
CaM	calmodulin
ECE	endothelin-converting enzyme
GSK3	glycogen synthase kinase 3
IAPP	islet amyloid polypeptide
MHC	major histocompatibility complex
NP2	neprilysin-like endopeptidase
PrP^C^	cellular prion protein
SST	somatostatin
STIM1	stromal interaction molecule 1
